# Catheter-Directed Vegetation Aspiration in Tricuspid Valve Bacterial Endocarditis: A Potential Treatment for Poor Surgical Candidates

**DOI:** 10.7759/cureus.24417

**Published:** 2022-04-23

**Authors:** Kyle Kelschenbach, Priya Patel, Michael Mamone

**Affiliations:** 1 Internal Medicine, Florida Atlantic University, Boca Raton, USA

**Keywords:** angiojet, angiovac, enterococcus faecalis, vegetation aspiration, septic emboli, tricuspid endocarditis, bacterial endocarditis

## Abstract

Infective endocarditis is a condition that has the potential to cause significant morbidity and mortality. Potential complications include sepsis, heart failure, atrioventricular block, embolic stroke, septic emboli, and intracardiac abscess formation. The backbone of treatment is intravenous antibiotics; however, in certain clinical scenarios, surgical management is also indicated to reduce complications and mortality. There exists a challenging subset of patients who require surgery but carry a high perioperative mortality risk. Percutaneous management of endocarditis is emerging as a potential treatment for this high-risk group of patients: it allows for an attempt at source control while avoiding the high risks of surgery. Herein, we present the case of a 35-year-old male presenting with hemoptysis secondary to pulmonary septic emboli in the setting of *Enterococcus faecalis *tricuspid endocarditis. He was determined to be a poor surgical candidate and underwent catheter-directed debulking of the tricuspid vegetation with excellent results.

## Introduction

Infective endocarditis is characterized by infection of the endocardial surface of the heart. Infection can manifest in multiple ways, including valvular vegetation, myopericarditis, and abscess formation [1]. Valvular vegetation can produce a multitude of complications in almost any organ system due to septic emboli, including the brain, lungs, and kidneys. Given the potential for catastrophic complications, the presence of recurrent septic emboli is an indication of surgical management. Other indications include, but are not limited to, heart failure, persistent bacteremia despite appropriate therapy, and heart block [1].

Bacterial endocarditis carries a significant risk for mortality, with some multicenter studies noting the risk to be 15-20% while in-hospital and up to 40% in one year [2]. The complications mentioned above significantly increase the risk of mortality, and therefore these patients require surgery to mitigate these risks. However, surgical management also has its own risk of mortality. Concerning the surgical treatment of right-sided endocarditis, one study of 910 patients found mortality of 6.3% in patients having a valve replacement, 7.6% in those having a valve repair, and 12% in those having a valvectomy [3]. Furthermore, some patients even carry a higher rate of mortality due to comorbid conditions or other reasons. These types of patients would benefit from alternative management that can better balance the risks and benefits.

Over the last 10 years, catheter-directed aspiration of right-sided vegetation has emerged as a potential treatment for patients who are deemed high risk for open surgical management. Several case reports and case series have demonstrated success with decreasing the size of vegetation while conferring less risk than surgery due to a percutaneous approach. Our case involves a 35-year-old male who underwent successful percutaneous debulking of tricuspid vegetation due to being a poor surgical candidate. These facts and our case highlight the need to further optimize the management of bacterial endocarditis and to establish concise guidelines regarding the surgical and catheter-based treatment options.

This article has been accepted to be presented as a digital poster at the 2022 Society of Hospital Medicine Florida Chapter Summit Virtual Scientific Abstract Competition.

## Case presentation

A 35-year-old male with a medical history of asthma, morbid obesity, and type 2 diabetes presented to our emergency room with hemoptysis that began two days prior. He described expectorating thick red blood with clots and no other sputum. He also reported intermittent shortness of breath but denied fever, palpitations, or chest pain. The patient endorsed a history of IV drug use five years before but denied current use. He denied recent travel or exposure to incarcerated and homeless individuals.

Initial vital signs revealed a blood pressure of 177/100, a heart rate of 143, and an oxygen saturation of 78% breathing ambient air. Physical examination showed diaphoresis and morbid obesity but was otherwise unremarkable for murmurs, adventitious lung sounds, or vascular and immunological phenomena. Abnormalities on a complete blood count included a leukocytosis of 12,000 and a hemoglobin of 10.2 g/dL, and abnormalities on a basic metabolic profile revealed a sodium level of 127 mEq/L and a blood glucose level of 440 mg/dL. A hemoglobin A1c was 13.1.

**Figure 1:**
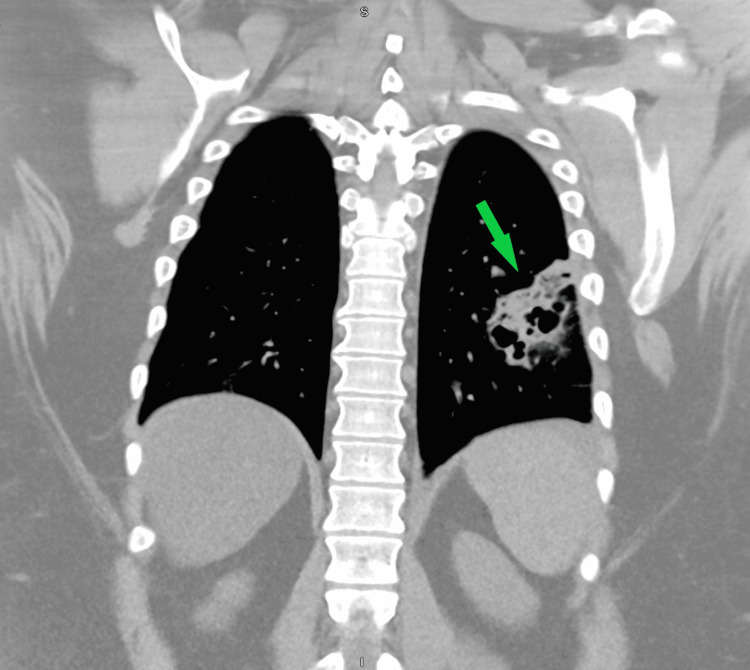
CT of the chest showing multiple cavitary lesions in the left lung consistent with pulmonary septic emboli CT, computed tomography.

A chest X-ray showed lower lobe predominant pulmonary interstitial changes that were slightly asymmetric on the left. A CT scan of the chest without contrast was obtained for further evaluation, which demonstrated multifocal consolidations and patchy opacities with multiple cavitary lesions, particularly in the left lower and left upper lobes, concerning a necrotizing or granulomatous process (Figure 1). The patient was initially started on vancomycin and cefepime and was placed on airborne precautions due to the possibility of pulmonary tuberculosis.

**Figure 2:**
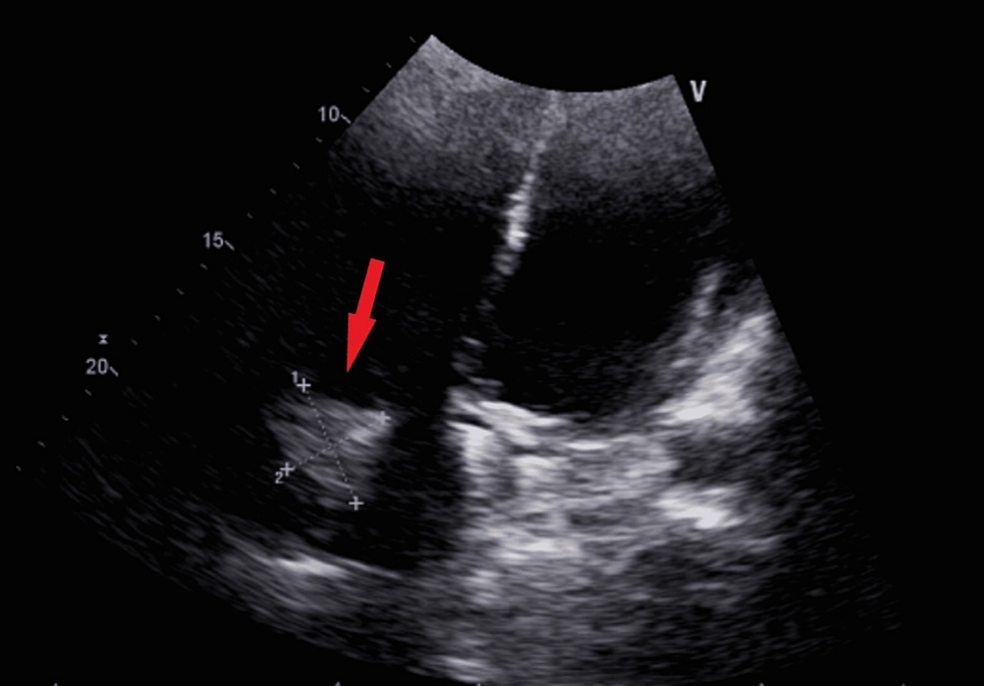
Transthoracic echocardiography showing a 3.8 cm $\times$ 3.2 cm globular vegetation on the tricuspid valve

Blood cultures obtained ultimately grew *Enterococcus faecalis*, and therefore a transthoracic echocardiogram was performed due to suspicion of infective endocarditis. This revealed a large 3.8 cm$\times$3.2 cm vegetation on the tricuspid valve with moderate tricuspid regurgitation (Figure 2). His antibiotics were then adjusted to ampicillin and ceftriaxone based on blood culture sensitivities. Given the large size of his vegetation and his pulmonary septic emboli, he was evaluated by a cardiothoracic surgeon for possible open surgical management. However, due to his morbid obesity and uncontrolled diabetes, he was deemed a poor surgical candidate. After a multidisciplinary discussion between cardiology, cardiothoracic surgery, and interventional radiology (IR), he underwent catheter-directed transthoracic echocardiogram and fluoroscopic-guided aspiration of the vegetation with IR using a 12 French aspiration catheter. This resulted in debulking of the vegetation to approximately 1.5 cm in size. The patient experienced approximately 500 cc of blood loss during the procedure and required transfusion of two units of packed red blood cells, but otherwise tolerated the procedure and remained hemodynamically stable.

Due to his history of IV drug use, the patient chose to remain inpatient for the full duration of his antibiotic treatment instead of being discharged and returning daily for intravenous therapy. He went on to receive six weeks of ampicillin 2 g every 6 h as well as ceftriaxone 2 g every 12 h, and the rest of his hospital course was normal. A repeat transthoracic echocardiography (TTE) was performed before discharge and showed a 1.4 cm$\times$0.9 cm vegetation on the tricuspid valve with moderate regurgitation. Unfortunately, the patient was then lost to follow-up.

## Discussion

Due to the possibility of significant morbidity and mortality, the rapid diagnosis and effective treatment of bacterial endocarditis is imperative. In 2010, there were 1.58 million disability-adjusted life-years or years of healthy life lost as a result of death and nonfatal illness or impairment secondary to infective endocarditis [4]. Although the prompt initiation of antibiotics can decrease the risk for complications and reduce mortality, a subset of patients require surgical management to improve their prognosis. Our patient emphasizes the challenging situation that can arise when a patient requires surgery but has a high perioperative risk due to comorbidities. Catheter-directed aspiration and debulking of valvular vegetation is emerging as a therapy for patients who need more advanced therapy but are considered poor surgical candidates.

In 2013, Divekar et al. described the first report of percutaneous aspiration of bacterial vegetation on a bioprosthetic pulmonary valve in a 17-year-old male. The AngioVac aspiration system was employed, which successfully debulked the vegetation and resulted in the clearance of blood cultures [5]. Since then, the cases and evidence supporting catheter-directed vegetation aspiration are limited but growing. Several reports have demonstrated the success and tolerability of this type of procedure: both the AngioJet rheolytic catheter system and the AngioVac straight catheter have been shown to reduce vegetation size by more than 50% [6,7].

One of the largest retrospective studies done to date was by George et al. in 2017, in which the authors examined 33 cases for percutaneous management of tricuspid valve endocarditis. They documented an average vegetation size of 2.1$\pm$0.7 cm with a 61% reduction in size following aspiration. All patients included in the study survived the procedure and 90.9% survived the index hospitalization [8].

Although percutaneous management of endocarditis is attractive, there are notable risks associated with this route as well. Our patient suffered 500 cc of blood loss during the procedure and required the transfusion of two units of red blood cells. Other risks include fragmentation of vegetation into the pulmonary circulation, as well as the possibility of hemolysis leading to renal failure [6]. These risks must be taken into account when performing a comprehensive analysis of each patient’s condition to make an informed decision regarding therapy. The risks and benefits of each treatment option must be carefully considered, ideally by a multidisciplinary team consisting of a general cardiologist, cardiothoracic surgeon, infectious disease specialists, and an interventional radiologist or interventional cardiologist, if indicated.

One limitation to our case and discussion is the lack of long-term follow-up with our patient. Although he was discharged in a stable condition, long-term follow-up on his outcomes would have helped support this method for treatment of right-sided valvular vegetation.

## Conclusions

Catheter-directed aspiration or debulking of tricuspid valve vegetation is emerging as an efficacious option for patients that require surgical management but have a high perioperative risk. This method offers a minimally invasive approach for source control that can help reduce or prevent complications caused by valvular vegetation. Our cases and those discussed above suggest that this approach can have favorable outcomes. That being said, cautious optimism is required, as current data are limited and this procedure does carry risks of its own. Given that bacterial endocarditis remains a prevalent condition with the potential for high morbidity and mortality, further studies and evidence are required to assist in determining the best possible treatment for this condition.
